# Effectiveness of FIBEROPTIC phototherapy compared to conventional phototherapy in treating HYPERBILIRUBINEMIA amongst term neonates: a randomized controlled trial

**DOI:** 10.1186/s12887-020-02458-2

**Published:** 2021-01-11

**Authors:** Helvi N. Joel, Deborah N. Mchaile, Rune N. Philemon, Ronald M. Mbwasi, Levina Msuya

**Affiliations:** 1 Department of Paediatrics and Child Health, Kilimanjaro Christian Medical University College, P O Box 2240, Moshi, Tanzania; 2 Department of Paediatrics and Child Health, Kilimanjaro Christian Medical Centre, P O Box 3010, Moshi, Tanzania

**Keywords:** Phototherapy, Unconjugated hyperbilirubinemia

## Abstract

**Background:**

Neonatal jaundice is one of the most common problems in neonates. Effective treatment of jaundice requires therapeutic intervention with high quality phototherapy. Over recent years, several studies reported fiberoptic phototherapy to be less effective than conventional phototherapy in term neonates. Our study aimed to compare the effectiveness of fiberoptic phototherapy with a larger illuminated area and higher irradiance to conventional phototherapy methods.

**Methods:**

This was a randomized controlled trial conducted at the Kilimanjaro Christian Medical Centre (KCMC). A total of 41 term neonates, less than 7 days of age with unconjugated hyperbilirubinemia were randomized. Thirteen (13) neonates were allocated to receive fiberoptic phototherapy, 13 to blue light conventional phototherapy and 15 to white light conventional phototherapy. Effectiveness was assessed by comparing the duration of phototherapy, bilirubin reduction rate and side effects of treatment. The data was analyzed with the independent t-test.

**Results:**

The mean overall bilirubin reduction rate was comparable in the fiberoptic phototherapy group (0.74%/h) and the blue light conventional phototherapy group (0.84%/h), with no statistically significant difference (*p*-value 0.124). However, white light conventional phototherapy had a significantly lower mean overall bilirubin reduction rate (0.29%/h) as compared to fiberoptic phototherapy (*p*-value < 0.001). The mean treatment duration of phototherapy was 69 h, 68 h and 90 h in the fiberoptic, blue light conventional and white light conventional phototherapy groups respectively. Side effects such as loose stool and skin rash were noted in some participants who received conventional phototherapy. No side effects of treatment were noted in the fiberoptic phototherapy group.

**Conclusion:**

The effectiveness of fiberoptic PT and blue light conventional PT were comparable in terms of bilirubin reduction rate and treatment duration, whereas fiberoptic phototherapy was more effective than white light conventional PT, with a significantly higher bilirubin reduction rate and shorter treatment duration. Fiberoptic phototherapy may mitigate side effects caused by conventional phototherapy.

**Trial registration:**

The Pan African Clinical Trial Registry, PACTR202004723570110. Registered 22nd April 2020- Retrospectively registered.

## Background

Globally an estimated 50% of term neonates develop hyperbilirubinemia (which may manifest as jaundice), typically 2–4 days after birth. About 25% of these babies will require phototherapy (PT) to avoid the effect of high serum unconjugated bilirubin, which can lead to bilirubin-induced neurologic dysfunction (BIND). BIND occurs when bilirubin crosses the blood-brain barrier and binds to brain tissue, resulting in brain injury if not treated appropriately in a timely fashion [1–3].

Unconjugated hyperbilirubinemia in the neonate is typically caused by the normal physiological inability of the neonate to process bilirubin adequately due to the combined effects of increased red blood cell turnover and a transient deficit in bilirubin conjugation in the liver [3, 4]. Moreover, it can also occur from underlying pathological conditions which result in increased bilirubin production and/or decreased bilirubin excretion [5], these include hemolytic disease of the newborn, polycythemia, extravasation of blood, sepsis with disseminated intravascular coagulation, increased enterohepatic circulation, inborn errors of metabolism and metabolic disorders [6].

Two interventions, phototherapy and exchange transfusion, are used to reduce total serum bilirubin (TSB) levels for infants with or at risk of developing hyperbilirubinemia. Phototherapy is now the preferred method of treatment for neonatal hyperbilirubinemia by virtue of its non-invasive nature and its safety [7–9], therefore, the use of high-quality phototherapy is paramount to decrease the likelihood of exchange blood transfusion.

Conventional and fiberoptic phototherapy have been proven to be equally effective in the treatment of hyperbilirubinemia among preterm neonates [10, 11]. However, a Cochrane review reported the efficacy of fiberoptic phototherapy in a number of different clinical situations and patient populations, and found that fiberoptic phototherapy was less effective than conventional phototherapy at lowering serum bilirubin in term neonates [11, 12]. These findings were attributed to the low irradiance and/or small surface area illuminated by the mat of the fiberoptic phototherapy units used in previous studies [3, 10, 12].

We hypothesize that increasing both the illuminated area and irradiance will improve the effectiveness of fiberoptic phototherapy with regard to bilirubin reduction rate and duration of treatment among term neonates with unconjugated hyperbilirubinemia. Improved effectiveness with fiberoptic phototherapy might mitigate the side effects of conventional phototherapy, create greater mother neonate bonding, encourage breastfeeding and obviates the need for eye patches.

Therefore, our study aimed to compare fiberoptic phototherapy with both a larger illuminated area and high irradiance to conventional phototherapy with regard to bilirubin reduction rate, short term side effects and duration of treatment among term neonates with unconjugated hyperbilirubinemia.

## Methods

### Study design, duration and study area

A parallel randomized controlled trial to test three treatment groups was conducted in the neonatal care unit (NCU) at the Kilimanjaro Christian Medical Centre (KCMC) in Northern Tanzania from January 2019 to May 2019. The NCU has a bed capacity of 62, with an average nurse to patient ratio of 1:10 per shift. The babies are nursed in locally made cots which are heated by incandescent bulbs. In our neonatal care unit, conventional phototherapy has been the only treatment modality used for unconjugated hyperbilirubinemia.

### Eligibility criteria

Sixty-five term neonates (> 37 weeks of gestation), less than 7 days of age, admitted with jaundice, were identified by clinicians working in the neonatal unit and screened for eligibility. We included those with both hemolytic and non-hemolytic unconjugated hyperbilirubinemia with a total serum bilirubin level that has reached phototherapy threshold values as per the American Academy of Pediatrics (AAP) nomogram which is a validated tool used for making decision regarding phototherapy in infants with unconjugated hyperbilirubinemia. The threshold for initiating phototherapy in term neonates with no risk factors (risk factors = isoimmune hemolytic disease, asphyxia, significant lethargy, temperature instability, sepsis) was the low risk line on the AAP nomogram. Whereas the threshold for initiating phototherapy in term neonates with a risk factor was the medium risk line on the AAP nomogram. Excluded from the study were: neonates receiving phenobarbitone, neonates with bilirubin levels that have reached exchange transfusion levels on the AAP nomogram, neonates with conjugated hyperbilirubinemia, neonates who have already received phototherapy prior to enrollment, and those whose parents refused to consent.

### Sample size estimation

In order to obtain our sample size, we estimated a 34 μmol/L effect size in the three arms, with a level of significance of 5%, power of 85% and an estimated SD of 24 μmol/L for the level of serum bilirubin after 24 h. The minimal expected sample size in addition to non-response rate of 20% was 39. We included in our final analysis a total of 41 term neonates who met the inclusion criteria.

### Study variables and data collection method

Written consent was obtained from parents of eligible participants. A questionnaire was used to collect demographic data such as sex, gestational age at birth (determined according to the maternal history and Ballard’s scoring system), mode of delivery, birth weight in kilograms, age at enrollment in days, and mode of feeding.

Simple randomization was performed by drawing a paper from a container containing 45 folded papers: 15 marked ‘FB’ (Fiberoptic BiliBlanket), 15 marked ‘B’ (Bluelight PT) and 15 marked ‘W’ (White light PT). Participants were then allocated to intervention groups by the research assistants. This was an open label trial whereby both the parent of the participants and the researchers knew which intervention the neonate was receiving.

Before phototherapy initiation, a clinical assessment was done on the neonate, and vitals were recorded. Laboratory characteristics such as total serum bilirubin and direct serum bilirubin were recorded. Phototherapy was employed as per our standard nursery protocol and equipment. The neonate was nursed in an open cot wearing only a diaper which was folded to allow maximum skin exposure to phototherapy, and was turned every 2 h from prone to supine positions.

The white light PT unit “Atom model PIT- 220 TL” (Atom Medical), consists of six white fluorescent bulbs (PHILIPS, TL-D 18 W/64–765) and was placed 35 cm above the neonate with an illuminated cabinet of 66.5 cm × 34.5 cm and a mean irradiance of 8 μW/cm2/nm. The blue light PT unit “Olympic Bili-Lite model 66” (Olympic Medical), consisted of four Olympic blue fluorescent bulbs placed 20 cm above the neonate with an illuminated cabinet of 66 cm × 27.9 cm and a mean irradiance of 27 μW/cm2/nm. Eye pads were used for neonate on conventional phototherapy to prevent damage to the retina. The BiliBlu LED fiberoptic phototherapy unit (Martand Medical Services) with a body surface area of 25 cm × 40 cm was wrapped around the neonate with a mean setting of 34 μW/cm^2^/nm irradiance. Irradiance of the phototherapy units was measured using the GE Healthcare BiliBlanket Light Meter II. Irradiance in all phototherapy units was measured from the skin surface of the neonate, 3 measurements were taken: one measurement centrally and two measurements at the two most extreme peripheries exposed to the PT light, subsequently an average irradiance was calculated for each participant. Phototherapy was administered continuously except during minor procedures such as feeding, diapering, physical examination and capillary blood sampling.

Serum bilirubin reduction rate was assessed as the decline in the TSB levels for the duration of exposure to phototherapy, expressed as a percentage of decline per hour. This was monitored through daily total serum bilirubin levels. Treatment failure was defined as the need for additional phototherapy units (‘double phototherapy’) determined by serial serum bilirubin response while on phototherapy; a rise in serum bilirubin level more than 9 μmol /L per hour after phototherapy initiation was an indicator for double phototherapy. Phototherapy was stopped when serum bilirubin levels were 50 μmol/L below the phototherapy threshold value on the AAP nomogram. Treatment duration was then assessed as the duration of time of exposure to phototherapy in hours.

Blood samples were analyzed at the KCMC clinical laboratory. The Cobas Integra 400 Plus (Roche Company) was used to analyze the serum bilirubin levels. The sample reached the laboratory within 15 min of collection. A minimum of 1 ml of blood was required. Care was taken to prevent exposure to light by placing the samples in a dark blood transportation box.

Neonates were closely monitored for short term side effects of treatment which included: loose, greenish stools; hydration status: assessed as the difference in daily body weight after starting PT (5% body weight loss was considered as mild dehydration, 5–10% body weight loss was considered as moderate dehydration and more than 10% body weight loss was considered as severe dehydration); skin rashes and brownish discoloration of skin assessed by a dermatologist. Participant’s vital signs including temperature, respiratory rate, heart rate were monitored every 4 h throughout phototherapy. Weight was checked daily.

Feeds were calculated based on the daily kilocalories requirement for age. At enrollment, mothers were asked to express breast milk to quantify the milk production. When necessary, neonates were supplemented with infant formula milk to maintain the total daily feeds requirement. Neonates were fed every 3 h in all PT groups. Those who were not maintaining adequate oral intake were supplemented with IV fluids to maintain the total daily fluid requirement, considering the postnatal age, and clinical and laboratory findings.

Research assistants recruited for the study were registered nurses working in the NCU. Research assistants enrolled the participants for the study. They were trained on how to randomize and assign participants to intervention. They were familiarized with the study protocol for PT, how to correctly place the PT unit, when to interrupt PT with clear documentation of duration of PT interruption, and measurement of the body weight & vital signs (axillary temperature, heart rate, respiratory rate). A pilot study was conducted to assess the feasibility of the fiberoptic PT unit and to familiarize the research assistants with the study.

### Data analysis

Data analysis was done using Statistical Package for Social Sciences (SPSS) version 23. Mean and standard deviation was used for descriptive statistics. The data was analyzed with the independent t-test to compare means of bilirubin reduction rate (%/hr) and treatment duration between the treatment groups. Analysis of variance was used to compare the baseline and clinical characteristics between the three treatment groups. A *p*-value of ≦0.05 was regarded as statistically significant.

## Results

### Participant flow

**Fig. 1:**
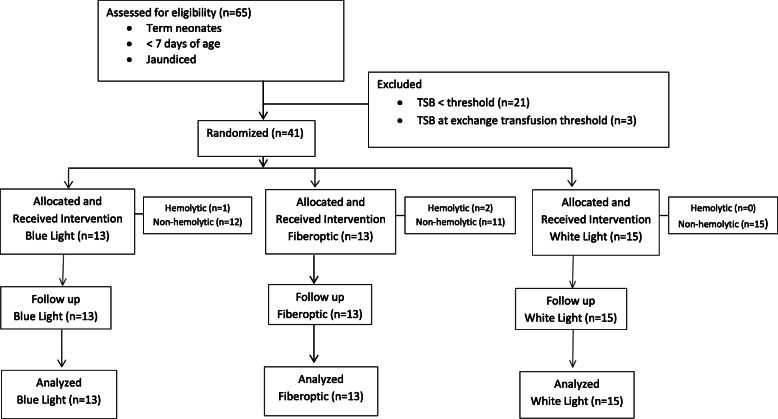
Participant flow chart. Flow chart showing the progress through the phases of a parallel randomized controlled trial of three groups: blue light conventional phototherapy, white light conventional phototherapy and fiberoptic phototherapy

A total of 65 term neonates, less than 7 days of age were admitted with jaundice between January 2019 to May 2019 and were assessed for eligibility. Of these, 24 participants were excluded: 21 had TSB levels below the phototherapy threshold and 3 had TSB levels at the exchange transfusion threshold. Forty-one participants were randomized and allocated to the three intervention groups. Thirteen 13 (31.7%) were allocated to blue light conventional phototherapy (1 participant with hemolytic jaundice and 12 participants with non-hemolytic jaundice), 15 (36.6%) were allocated to white light conventional phototherapy (all participants had non-hemolytic jaundice) and 13 (31.7%) were allocated to fiberoptic phototherapy (2 participants with hemolytic jaundice and 11 participants with non-hemolytic jaundice). All participants received the assigned intervention and were followed until phototherapy was stopped. Final analysis was performed on 41 participants as per allocation to intervention; no participants discontinued the study (Fig. 1).

### Baseline and clinical characteristics: comparison of the three treatment groups

The mean irradiance was significantly higher in the fiberoptic PT group (34.0 ± 1.24) as compared to the blue light and white light phototherapy groups (27.0 ± 1.08 and 8 ± 0.69 respectively) (*p*-value < 0.001). The mean gestation age at birth was 39 weeks for the white light conventional and fiberoptic PT groups and 38 weeks for the blue light conventional PT group (*p*-value 0.582). The mean birth weight was 2.9 kg in the blue light PT group, 3.1 kg in the white light PT group and 3.3 kg in the fiberoptic PT group (*p*-value 0.109). The mean age at onset of jaundice was 3 days across all 3 PT groups (*p*-value 0.829). The mean weight at enrolment and mean weight at the end of PT remained the same in all PT groups (2.7 kg, 3.2 kg and 2.9 kg in the blue light, fibreoptic and white light conventional PT respectively (*p*-value 0.086)). The mean axillary temperature during phototherapy was 37 °C across all the groups (*p*-value 0.937). The mean total serum bilirubin levels at the start of PT were comparable in all three groups with the highest being 299.75 in the fiberoptic PT group and the lowest being 283.71 in the white light PT group (*p*-value 0.843) (Table 1).

**Table 1:** Baseline and clinical characteristics: Comparison of the three treatment groups (*N* = 41)

Characteristics	Blue Light (***n*** = 13)	Fiberoptic (***n*** = 13)	White Light (***n*** = 15)	ρ value
	**Mean ± SD**	**Mean ± SD**	**Mean ± SD**	
Birth weight (Kg)	2.9 ± 0.4	3.3 ± 0.6	3.1 ± 0.5	0.109
Age at Enrolment (Days)	4 ± 1.0	4 ± 1.0	4 ± 1.0	0.318
Gestation age at birth in weeks	38 ± 2.0	39 ± 2.0	39 ± 1.0	0.582
Weight at enrolment	2.7 ± 0.3	3.2 ± 0.6	2.9 ± 0.5	0.086
Weight at end of PT	2.7 ± 0.3	3.2 ± 0.7	2.9 ± 0.5	0.131
Mean axillary temperature during phototherapy (°C)	37.04 ± 0.38	37.06 ± 0.40	37.09 ± 0.43	0.937
Age at onset of jaundice in days	3 ± 1.0	3 ± 1.0	3 ± 0.0	0.829
Serum bilirubin at start of phototherapy μmol/l	294.80 ± 78.49	299.75 ± 76.63	283.71 ± 69.52	0.843
Average irradiance (μW/cm2/nm)	27 ± 1.08	34 ± 1.24	8 ± 0.69	< 0.001^*^

Values presented as mean ± SD

*p*-value analyzed using analysis of variance

^*^ Significant *p*-value

### Total serum bilirubin reduction rate

#### Total serum bilirubin reduction rate per 24 h as a %/hour

The total serum bilirubin reduction rate (as %/hour) in the blue light conventional phototherapy was 0.56, 0.73, 0.89 and 1.21 for each subsequent 24 h. Whereas, the fiberoptic phototherapy total serum bilirubin reduction rate (as %/hour) was 0.59, 0.61, 0.76 and 0.99 for each subsequent 24 h. The white light phototherapy had the lowest total serum bilirubin reduction rate (as %/hour) which was 0.14, 0.22, 0.31, 0.45, and 0.30 on each subsequent day. There was no statistically significant difference of the total serum bilirubin reduction rate per 24 h between blue light conventional and fiberoptic PT, however there was statistically significant difference between fiberoptic and white light conventional PT (Table 2).

**Table 2:** Total serum bilirubin reduction rate per 24 h (as %/hour), Overall bilirubin reduction rate (as %/hour) and Mean treatment duration in hours (*N* = 41)

Variables	Fiberoptic (*n* = 13)	Blue Light (*n* = 13)	White Light (*n* = 15)	Mean difference (Fiberoptic vs Blue Light)	*p*-value	Mean difference (Fiberoptic vs White Light)	*p*-value
	Mean ± SD	Mean ± SD	Mean ± SD				
Total serum bilirubin reduction rate per 24 h, %/hour							
0-24 h	0.59 ± 0.18	0.56 ± 0.13	0.14 ± 0.07	0.03 ± 0.06	0.727	0.45 ± 0.05	< 0.001**
24-48 h	0.61 ± 0.23	0.73 ± 0.17	0.22 ± 0.06	0.12 ± 0.08	0.065	0.39 ± 0.06	0.006**
48-72 h	0.76 ± 0.23	0.89 ± 0.15	0.31 ± 0.12	0.13 ± 0.08	0.053	0.45 ± 0.07	0.034**
72-96 h	0.99*	1.21*	0.45 ± 0.17	0.78	–	0.54 ± 0.18	–
96-120 h	–	–	0.30*	–	–	–	–
Overall bilirubin reduction rate, %/hour Mean treatment duration in hours	0.74 ± 0.19	0.84 ± 0.40	0.28 ± 0.12	0.10 ± 3.28	0.125	0.46 ± 2.70	0.008**
	69.46 ± 7.55	68.77 ± 11.61	90.33 ± 19.91	0.69 ± 3.84	0.858	20.87 ± 5.19	< 0.001**

Values presented as mean ± SD

*p*-value analyzed using independent t-test

^−^ no value computed, *Remained with few participants hence SD could not be estimated

** Significant *p*-values

#### Overall total serum bilirubin reduction rate as %/hr.

On average, blue light conventional PT reduced bilirubin levels at a rate of 0.84% per hour, which was the highest overall reduction rate, followed by fiberoptic phototherapy at 0.74% per hour and the lowest overall bilirubin reduction rate was in the white light conventional PT group at 0.29% per hour. There was statistically significant difference in the overall bilirubin reduction rate between the fiberoptic and white light conventional PT group (*p*-value < 0.001). However, there was no statistically significant difference when we compared the overall bilirubin reduction rate between blue light conventional and fiberoptic PT (*p*-value 0.124) (Table 2).

### Treatment duration

#### Mean treatment duration

The mean treatment duration in hours was similar in the blue light PT group and fiberoptic PT group (68 vs. 69 h, *p*-value 0.858). However, the mean treatment duration was longer in the white light conventional PT group (90 h) than in the fiberoptic PT group (*p*-value 0.002) (Table 2).

#### Side effects of treatment

Side effects such as loose stool (blue light PT group: 38%, white light PT group: 20%) and transient maculopapular rashes (Blue light PT group: 15%) were noted in the conventional PT groups. No side effects were noted in the fiberoptic PT group.

## Discussion

In the present study, phototherapy was effective in decreasing bilirubin levels in all three groups. The response was greater in the blue light conventional phototherapy group (0.84%/h), followed by fiberoptic phototherapy (0.74%/h), whereas the white light conventional phototherapy (0.29%/h) had the lowest response in lowering serum bilirubin levels. The effectiveness of fiberoptic PT and blue light conventional PT were comparable in terms of bilirubin reduction rate and treatment duration, whereas fiberoptic phototherapy was more effective than white light conventional PT, with a significantly higher bilirubin reduction rate and shorter treatment duration. No side effects were reported in the fiberoptic PT group, while both conventional PT groups had participants with loose stool. Transient erythematous skin rash was noted in some patients receiving blue light conventional PT.

In this study, the effectiveness of fiberoptic PT and blue light conventional PT were comparable in terms of bilirubin reduction rate, whereas white light conventional PT had a significantly lower bilirubin reduction rate as compared to fiberoptic PT. These findings could be attributed to the distance of the light source from patient, surface area covered, wavelength and the irradiance of the phototherapy units [13]. Schuman et al. compared continuous fiberoptic PT to conventional PT comprised of both blue and white bulbs in the home setting [14, 15]. They found no statistically significant difference in the mean bilirubin reduction rate. The combination of blue and white light in the conventional PT unit used in Schuman et al. study may have influenced the wavelength of their conventional PT unit. One of the advantages of fiberoptic phototherapy is no interruption of exposure, which impedes separation of mother and baby and encourages breastfeeding with long duration of phototherapy. We therefore postulate that continuous PT could further improve the effectiveness of the fiberoptic PT unit as noted in the study by Schuman et al. Though not assessed in our study, we hypothesize that patients with physiologic jaundice, who are otherwise healthy, could receive PT at home, which would reduce hospitalized neonates, positively affecting the workload in the NCU and economic burdens to families and the hospital.

White light conventional PT had the lowest response in lowering serum bilirubin levels in this trial. In light of these findings, the use of white light conventional PT may be inadequate in term neonates with rapidly increasing TSB, especially of a hemolytic nature. A study by Tan increased the irradiance of the fiberoptic PT unit with the aim of improving the efficacy of the fiberoptic PT unit, however maintained a small illuminated surface area in their fiberoptic PT unit [15, 16]. This was compared to conventional PT using white light, and combined phototherapy which consisted of fiberoptic PT and white light conventional PT. Contrary to our findings, the efficacy of fiberoptic PT was distinctly less, with an overall mean decline rate of 0.49%/h, as compared to 0.70%/h in the white light conventional PT. The best result was obtained by combination exposure (0.97%/h). The latter findings could be explained by the surface area covered, improved wavelength spectrum and a higher spectral irradiance in this arrangement, being equal to the sum of the two forms of phototherapy. These findings suggest that combined phototherapy might be the best way to lower serum bilirubin levels in term neonates receiving white light conventional PT.

Our findings demonstrate that the effectiveness of fiberoptic PT and blue light conventional PT were comparable in terms of bilirubin reduction rate. This was in contrast to Sarici’s findings, who compared the efficacy of fiberoptic PT to conventional PT using blue light in healthy term neonates with non-hemolytic unconjugated hyperbilirubinemia [16, 18]. In their study, they increased the illuminated area of their fiberoptic PT unit with the aim of improving its efficacy. The lower bilirubin reduction rate of the fiberoptic phototherapy in their study may be due to its lower spectral irradiance. We deduce that the improved effectiveness noted in the fiberoptic PT group in our study could be explained by the joint effects of a larger illuminated area, higher irradiance and the LED lights in the blue wavelength spectrum, identical to the maximum bilirubin absorption spectrum. A phototherapeutic effect is seen only when the wavelength can penetrate tissue and absorb bilirubin [13]. It is noteworthy in Sarici’s study, that the participants in the fiberoptic PT group were slightly older in age as compared to those in the conventional PT group; as such skin maturity could have influenced their findings.

The treatment duration in studies by Tan and Sarici was longer in the fiberoptic PT group as compared to the conventional PT group [15, 16, 18]. This could be explained by the wavelength, surface area exposed and irradiance in the two different treatment modalities. Contrarily in our study, the treatment duration was comparable in the fiberoptic PT group and blue light PT group, and was significantly longer in the white light PT group. We therefore recommend fiberoptic phototherapy and blue light conventional PT as the treatment of choice, over white light conventional PT which has 1.3 times longer treatment duration. The longer treatment duration might increase the risk of acquiring nosocomial infection and increase the length of hospital stay, which has financial implications to both the hospital and family and also increases workload in the NCU.

Holtrop, Tan and Al-Alaiyan reported no side effects of treatment in the fiberoptic phototherapy group [16, 17, 19]. This was consistent with our findings. Studies by Sarici, Rouf et al. and Gutta et al. observed a significantly higher incidence of skin rashes and loose stools in the conventional phototherapy group which was similar to our findings [18–20]. In our study, participants in the blue light conventional PT group had the highest incidence of side effects. Based on these findings, the fiberoptic PT could be considered the treatment of choice to avoid side effects [18].

The AAP noted that the most significant bilirubin decline is considered in the first 24 h. The AAP further states that with the standard phototherapy units, a decrease of 6 to 20% of the initial bilirubin level can be expected in the first 24 h, which our fiberoptic phototherapy achieved with the highest bilirubin reduction rate of 0.59%/hour (equivalent to 14%) in the first 24 h [13, 14].

Effective treatment to decrease bilirubin levels in neonates with severe jaundice includes phototherapy and exchange transfusion. In our NCU, conventional phototherapy has been the only treatment modality used. Our study was an effectiveness study; the intent was to compare fiberoptic PT to white and blue light conventional PT as we typically use it in our neonatal unit, and hence we included neonates with both physiologic and pathologic jaundice. Previous studies were efficacy studies, which required substantial deviations from clinical practice. The studies by Holtrop and Tan excluded neonates with hemolytic jaundice, whereas Sarici excluded neonates with hemolytic jaundice, infections, congenital malformations and enclosed hematoma [16, 18, 19]. The major limitations of our study could be the use of suboptimal conventional phototherapy in the white light conventional phototherapy group as well as a relatively small sample size which could make generalization of results difficult.

## Conclusions

Increasing both irradiance and the illumination area of the fiberoptic PT unit improved effectiveness of phototherapy in term neonates with unconjugated hyperbilirubinemia. We therefore conclude that phototherapy delivered by the fiberoptic phototherapy unit is safe and the effectiveness is comparable to that of blue light conventional phototherapy, providing a convenient alternative phototherapy application strategy that obviates the need for eye patches and reduces side effects of treatment. It would likely also have favorable implications on patient costs and NCU workload if successfully adopted.

We recommend further studies to explore the feasibility of home phototherapy in our setting. In addition, we recommend studies to assess long term side-effects of the different phototherapies.

## Data Availability

The datasets used and analyzed during the current study are available from the corresponding author on reasonable request.

## Abbreviations

AAP: American Academy of Pediatrics
BIND: Bilirubin Induced Neurologic Dysfunction
KCMC: Kilimanjaro Christian Medical Centre
LED: Light Emitting Diode
NCU: Neonatal Care Unit
PT: Phototherapy
SVD: Spontaneous Vertex Delivery
TSB: Total Serum Bilirubin
